# Beyond protected areas: The importance of mixed‐use landscapes for the conservation of Sumatran elephants (*Elephas maximus sumatranus*)

**DOI:** 10.1002/ece3.10560

**Published:** 2023-09-28

**Authors:** Muhammad Ali Imron, Danielle M. Glass, Muhammad Tafrichan, Ramiro D. Crego, Jared A. Stabach, Peter Leimgruber

**Affiliations:** ^1^ Universitas Gadjah Mada Yogyakarta Indonesia; ^2^ Smithsonian National Zoo & Conservation Biology Institute Conservation Ecology Center Front Royal Virginia USA; ^3^ School of Biological, Earth & Environmental Sciences University College Cork Cork Ireland

**Keywords:** connectivity, *Elephas maximus sumatranus*, habitat suitability, human activity, species distribution modeling, *sumatra*, Sumatra Asian elephant

## Abstract

Elephants were once widely distributed across the Indonesian island of Sumatra but now exist in small, isolated populations. Using the best data available on elephant occurrence, we aimed to (a) predict potential habitat suitability for elephants (*Elephas maximus sumatranus*) across the island of Sumatra and (b) model landscape connectivity among the extant elephant populations. We used direct sightings and indirect observations of elephant signs, as well as six remotely sensed proxies of surface ruggedness, vegetation productivity and structure, and human land use and disturbance, to model habitat suitability in a Google Earth Engine (GEE) environment. We validated the habitat suitability prediction using 10‐fold spatial block cross validation and by calculating the area under the precision‐recall curve (AUC‐PR), sensitivity, and specificity for each model iteration. We also used a geolocation dataset collected from global positioning system (GPS) collars fitted on elephants as an independent validation dataset. Models showed good predictive performance with a mean AUC‐PR of 0.73, sensitivity of 0.76, and specificity of 0.68. Greater than 83% of the independent GPS collar geolocations were located in predicted suitable habitat. We found human modification, surface ruggedness, and normalized difference vegetation index to be the most important variables for predicting suitable elephant habitat. Thirty‐two percent, or 135,646 km^2^, of Sumatra's land area was predicted to be suitable habitat, with 43 patches of suitable habitat located across Sumatra. Areas with high connectivity were concentrated in the Riau and North Sumatra provinces. Though our analysis highlights the need to improve the quality of data collected on Sumatran elephants, more suitable habitat remains on Sumatra than is used by known populations. Targeted habitat conservation, especially of the suitable habitat in and around the Lamno, Balai Raja, Tesso Tenggara, Tesso Utara, Bukit Tigapuluh, Seblat, Padang Sugihan, and Bukit Barisan Selatan ranges, may improve the long‐term viability of this critically endangered species.

## Graphical abstract

Elephants (*Elephas maximus sumatranus*) were once widely distributed across the Indonesian island of Sumatra but now exist in small, isolated populations. We used random forest modeling in a Google Earth Engine (GEE) environment to model habitat suitability. Our analysis suggests that more suitable habitat remains on Sumatra than is used by the known populations, and that targeted habitat conservation may improve the long‐term viability of the critically endangered subspecies.

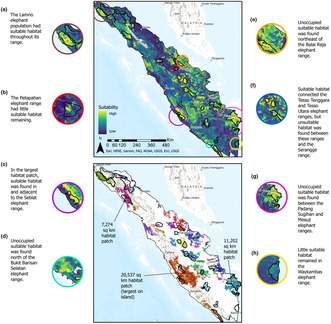

## 1 INTRODUCTION

Habitat suitability is a critical concept in biodiversity conservation and is especially important for species residing in areas undergoing rapid anthropogenic and ecological change (Durán et al., 2020). However, obtaining reliable information on habitat suitability, especially for endangered species, can be challenging because of limited data availability at broad spatial scales (e.g., regions, countries, or continents; Imron et al., 2016; Marcer et al., 2013; Sodik et al., 2020). Models provide important information about the physical space a species occupies in relation to the environmental conditions and resources that are available (Hall et al., 1997; Leopold, 1933), which can facilitate assessments of the impacts of environmental change on species distributions (e.g., land‐use change, climate change; Sinclair et al., 2010) and provide the relevant information to develop conservation management plans (Guisan et al., 2013).

Sumatra is one of the five largest islands in Indonesia (429,545 km^2^) and plays a pivotal role for biodiversity conservation across the region (Myers et al., 2000; Verma et al., 2020). Once considered a stronghold for the conservation of endangered species due to limited development pressure, Sumatra has experienced dramatic declines in the extent of its primary forest over the last three decades (Margono et al., 2012, 2014). Importantly, the loss and degradation of available forest has affected areas identified as primary habitat for a variety of endangered species, including keystone species such as Sumatran elephants (*Elephas maximus sumatranus)* and Sumatran tigers (*Panthera tigris sondaica*) (Imron et al., 2011; Moßbrucker et al., 2016; Widodo et al., 2017, 2022).

**FIGURE 1:**
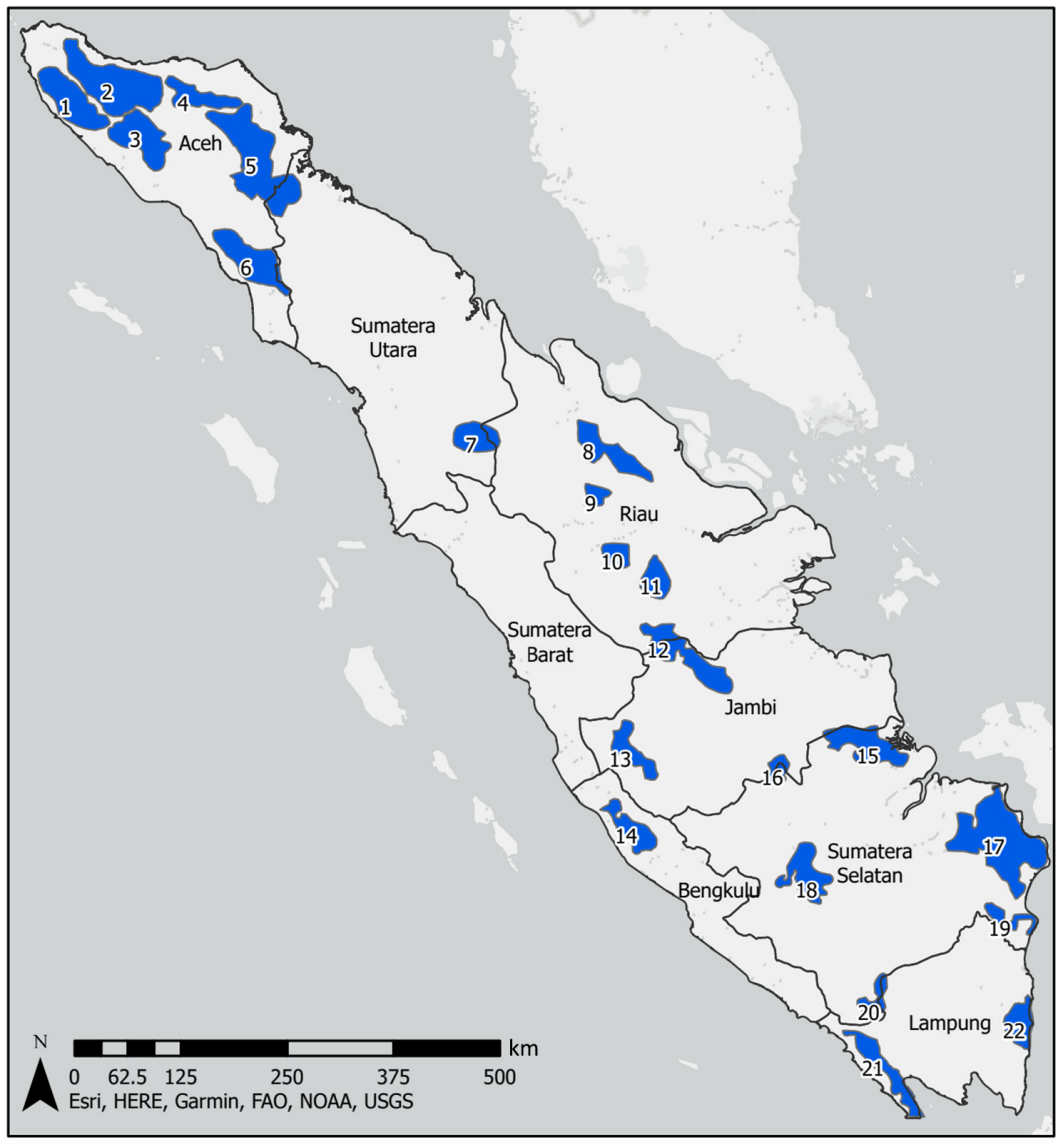
Elephant ranges on Sumatra with at least one animal present in 2020, as described by the Indonesian Ministry of Environment & Forestry and adapted by Moßbrucker (2021). To see the information about each population that corresponds to the numbers shown in the figure, see Table 1.

Sumatran elephants are the largest mammals found on Sumatra. Historically, elephants were distributed across the entire island with the exception of the steepest and most rugged locations (Jackson, 1990). In the 1980s, a rapid assessment survey estimated 2800–4800 individuals within 44 known elephant ranges (Blouch & Haryanto., 1984; Blouch & Simbolon, 1985). Despite bans on hunting, elephant populations halved between 1985 and 1999 due to continued habitat destruction and fragmentation (Katugaha et al., 1999; Santiapillai & Jackson, 1990). By 2011, nearly 70% of the species' potential habitat was lost in a single elephant generation, resulting in the International Union for Conservation of Nature (IUCN) listing the Sumatran elephant as “Critically Endangered” (Gopala et al., 2011). The elephant population at this time was estimated to be just 2400–2800 individuals. In 2020, the Indonesian Ministry of Environment & Forestry estimated the total elephant population to be 924–1359 individuals residing in 22 known elephant ranges (Moßbrucker, 2021; Figure 1, Table 1). Based on these assessments, only about 12% (49,733 km^2^) of Sumatra's terrestrial land surface may support elephants (Moßbrucker, 2021) and most of these locations represent island habitats with limited functional connectivity (A. Moßbrucker, pers. comm.).

**TABLE 1:** Estimated habitat area and elephant population size for 22 elephant ranges on Sumatra.

ID	Population name	Area (km^2^)	Population size
1	Lamno—Kreung Sabee—Teunom—Woyla Barat	283.270	65–80
2	Seulawah—Jantho (Simueleu)—Kemala—Tangse	493.433	40–50
3	Woyla Timur—Sungai Emas—Pante Cermin—Beutong	255.138	58–71
4	Alue Buloh—Cot Girek—Gereudong—Paya Bakong	139.242	25–35
5	Jambo Aye—Langkahan—Samarkilang—Lokop—Pinding—Kappi	580.984a	150–155
6	Klueut—Trumon—Bengkung—Subulussalam—Sultan Daulat	249.131	54–60
7	Mahato	152.905	3–10
8	Balai Raja; Rangau—Giam Siak Kecil	212.192	39–60
9	Petapahan	53.944	22–27
10	Tesso Tenggara	82.658	35–50
11	Tesso Utara; Tesso Selatan	110.749	102–151
12	Bukit Tigapuluh (Serangge)	281.048	150
13	Sipurak—Gunung Sumbing—Sungai Ipuh	142.078	20–29
14	Seblat—Air Teramang	144.518	6–10
15	Sungai Lalan	40.944	4–7
16	Hutan Harapan	60.704	8
17	Padang Sugihan—Lebong Hitam—Simpang Heran	639.681	15–32
18	Benakat—Semangus	269.765	28–32
19	Mesuji III	69.142	1–3
20	HPT. Saka—SM Gunung Raya	77.695	2–5
21	Bukit Barisan Selatan	195.627	88–219
22	Way Kambas National Park	139.187	144–225

*Note*: The elephant ranges are of at least one animal in 2020. This table was directly adapted from Moßbrucker (2021). The ID refers to locations shown in Figure 1.

Sumatran elephants have large habitat requirements, with home ranges estimated to be between 273 and 1352 km^2^ (Moßbrucker et al., 2016). Their presence is positively related to dense forest cover, although as a habitat generalist, Asian elephants can be found in open forest habitats, grassland, savannahs, mangroves, and low‐density agriculture (Fernando & Leimgruber, 2011; Leimgruber et al., 2011; Lin et al., 2008; Neupane et al., 2019; Songer et al., 2016; Varma, 2008). Habitat selection within specific ecosystems may be driven by forage availability, topographic factors, and the presence of humans (Calabrese et al., 2017; Moßbrucker et al., 2016). Human modification of the landscape generally degrades elephant habitat and lowers elephant use of the area (Chan et al., 2022; Jathanna et al., 2015; Kumar et al., 2015; Wall et al., 2021; Wilson et al., 2021). Efforts to conserve natural landscapes for wildlife have not accounted for elephant's habitat requirements, with only 12.3% of lands on Sumatra having a protected status (52,766.04 km^2^; Nurbaya et al., 2020). Further, most core elephant ranges are located outside of the protected areas network (Moßbrucker et al., 2016; Suhartono et al., 2007). Given the large area requirements of elephants, protected areas alone will likely not suffice to conserve and support substantial wild populations over multiple generations (Moßbrucker, 2021).

Despite calls for scientifically rigorous distribution and connectivity maps (Suhartono et al., 2007), no habitat suitability or cost of movement analyses have been conducted. Our aim was to use currently available data of elephant occurrence to (a) predict and identify potential habitat suitability for elephants across the island of Sumatra and (b) model landscape connectivity among the extant elephant populations. The information generated in our study will help to identify the remaining core elephant habitat, assess where elephant populations could expand, and prioritize important movement corridors. This range‐wide, spatial information can then be used to focus conservation efforts on the protection of the areas of Sumatra most critical for long‐term elephant persistence on the landscape.

## 2 METHODS

### 2.1 Data processing

**FIGURE 2:**
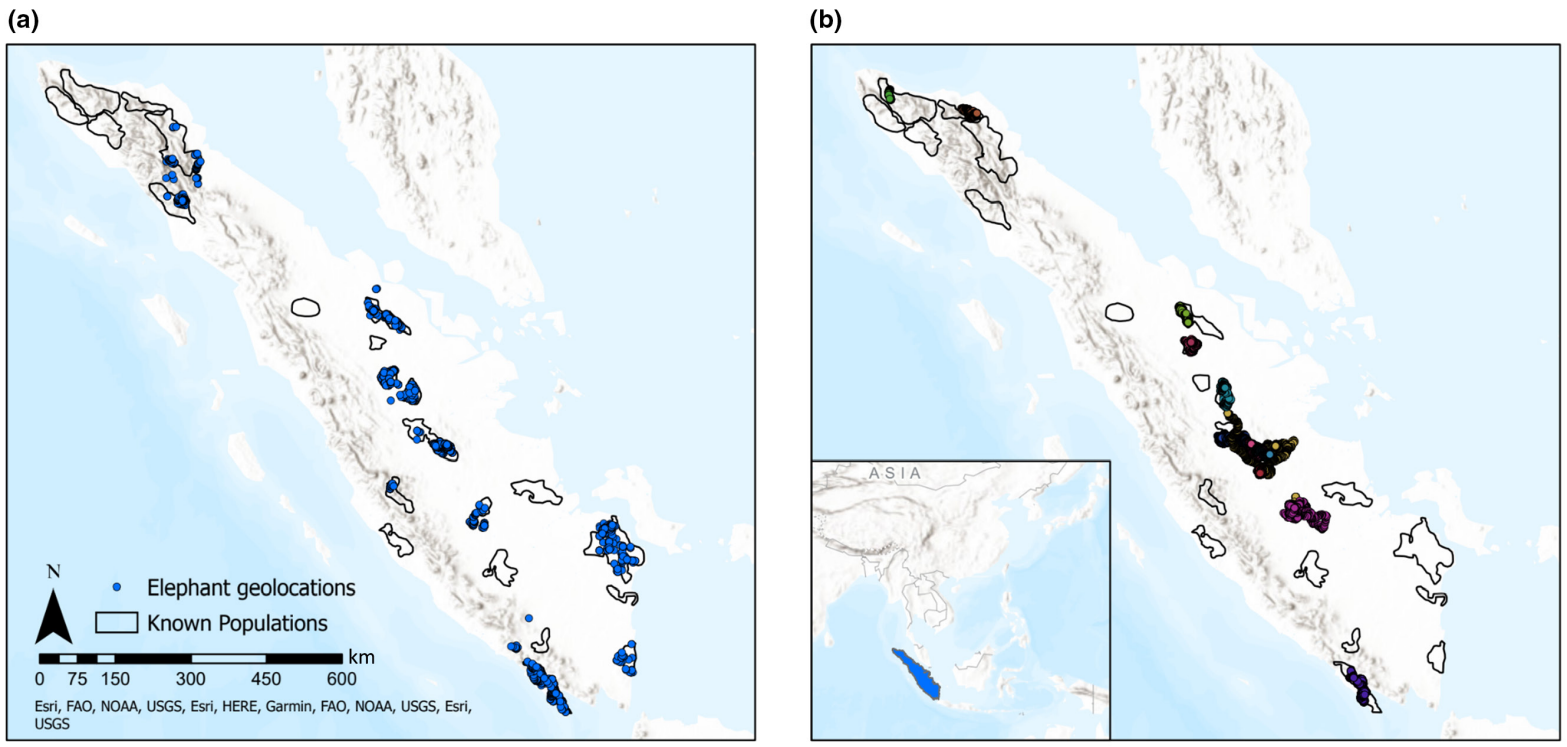
The observations of elephant occurrence used for model development (a) and the elephant GPS geolocation data used for model validation (b). The observations of elephant occurrence shown represent the dataset after the removal of potentially erroneous elephant observations, but prior to the thinning of the dataset to one geolocation/square kilometer. The GPS data map uses colors to differentiate the geolocations of each individual collared elephant. The known populations represent the elephant ranges of at least one animal in 2020.

We obtained 2952 observations of elephant occurrence on the island of Sumatra from the Indonesian Ministry of Environment and Forestry, including the Natural Resource Conservation Agencies of South Sumatra and Jambi provinces (see Table S1). Observations were either direct sightings or indirect observations of elephant signs (i.e., dung, scrapings, footprints, trails). All observations were collected between 2011 and 2020 during elephant population surveys, park ranger patrols, or investigations of human‐elephant conflict. Using high resolution satellite imagery in Google Earth Engine (GEE), we removed potentially erroneous occurrence observations that were located on top of buildings or in large water bodies. Our remaining occurrence dataset consisted of 2916 georeferenced locations (Figure 2a).

To understand how habitat availability influences Sumatran elephant distribution, we integrated six remotely sensed proxies of surface ruggedness, vegetation productivity and structure, and human land use and disturbance (Table 2). We derived surface ruggedness from 30 m Shuttle Radar Topography Mission (SRTM) elevation data (Farr et al., 2007), calculating the standard deviation of the data within a moving window, 500 m radius. Pixel neighborhoods with large standard deviations are rougher (i.e., have steeper slopes) than areas with smaller standard deviations (i.e., flat areas). We used normalized difference vegetation index (NDVI) data derived from the Moderate Resolution Imaging Spectroradiometer (MODIS; MOD13Q1, 250 m spatial resolution) as a proxy for primary productivity (Pettorelli et al., 2011). To characterize habitat structure, we utilized C‐band and L‐band synthetic aperture radar data (Shimada et al., 2014). In forests, these layers, respectively, denote the structure of the canopy and understory (Berninger et al., 2018; Omar et al., 2017; Thapa et al., 2013). We also incorporated the location of oil palm plantations, inclusive of year of establishment (Danylo et al., 2021; 30 m spatial resolution). We used the number of years since each pixel had been transformed from forest to palm oil as the predictor variable, assuming that selection by elephants changes throughout the economic lifespan of the oil palm plantation (Evans et al., 2017).

**TABLE 2:** Environmental layers used for Sumatran elephant habitat suitability modeling.

Data layer	Source	Resolution	Time period
Surface Ruggedness	Derived from the National Aeronautics and Space Administration Shuttle Radar Topography Mission's digital elevation data (Farr et al., 2007)	30 m	2/11/2000
NDVI	MOD13Q1.006 Terra Vegetation Indices 16‐Day Global 250 m (Didan, 2015)	250 m	1/1/13–12/2/2021
Palm Oil	Derived from Sentinel 1 imagery (Danylo et al., 2021)	30 m	Images collected 1/1/2017–12/31/2017
Human Modification	Derived from 13 data layers of anthropogenic activity including human settlement, agriculture, transportation, mining and energy production, and electrical infrastructure (Kennedy et al., 2019)	1 km	Given the 13 input data layers, the human modification layer most closely estimates the on‐the‐ground reality for the year 2016
Synthetic Radar Aperture C‐Band	Copernicus Sentinel‐1 Ground Range Detected, log scaling	10 m	1/1/13–12/2/2021
Synthetic Radar Aperture L‐Band	Global PALSAR‐2/PALSAR Yearly Mosaic (Shimada et al., 2014)	25 m	1/1/13–12/2/2021

*Note*: Time period refers to the temporal duration over which data was used in the model.

All predictor variables were resampled to 250 m resolution using the default nearest neighbor resampling method in GEE. To evaluate potential collinearity among environmental predictors, we estimated the Spearman correlation at 5000 random locations across Sumatra and calculated the variance inflation factor (VIF) of the predictor layers using the R package usdm (Naimi et al., 2014). We found no significant correlation between the covariates incorporated in the analyses (< 0.7 for all pairwise correlations; VIF <3 for all covariates).

### 2.2 Model development

To construct our elephant habitat suitability model, we implemented an SDM workflow in GEE (Crego et al., 2022; Gorelick et al., 2017). We choose to use GEE to implement the SDM due to the ease in accessibility of the desired raster products (i.e., SRTM, MODIS, Sentinel, Copernicus, PALSAR), availability of desired algorithms (i.e., Random Forest), high computing capacity, and the reproducibility of our modeling framework (see shared code). GEE utilizes a parallel computing system that improves efficiency by reducing computation time (Gorelick et al., 2017; Tamiminia et al., 2020).

To reduce the potential sampling bias of clustered presence locations that can lead to model overfitting and low model performance (Boria et al., 2014; Fourcade et al., 2014; Veloz, 2009), we randomly thinned the observational data to one location per square kilometer (*n* = 1167). In our modeling framework, we used random forest classifiers and a repeated (10‐fold) spatial block cross validation approach (Roberts et al., 2017; Valavi et al., 2022). From the different machine learning methods available in GEE, we chose random forests because it has been shown to commonly outperform other classifier algorithms (Crimmins et al., 2013; Hao et al., 2019, 2020; Valavi et al., 2022). Random forest is a supervised machine learning algorithm that functions by constructing a collection, or “forest,” of decision trees and selecting the result that is supported by the largest number of trees (Breiman, 2001). The independent data is bootstrapped and only a random subset of the dependent variables is considered at each step of the decision tree (Breiman, 2001; Schonlau & Zou, 2020). For cross validation, we defined 50 × 50 km blocks that were randomly split 10 times, 70% were used for model training and 30% for model validation, to ensure spatial independence between training and validation datasets (Roberts et al., 2017; Valavi et al., 2022). We created blocks across the entire Sumatra Island since elephants once ranged across its entire extent (Jackson, 1990).

At each model iteration, a set of presence points from the training block set was selected, and an equal number of pseudo‐absences were created randomly within the area of these blocks but at distances >1 km from any occurrence point. The >1 km distance limitation was chosen to correspond to the 1 km thinning of the observational data and ensure that pseudo‐absences were not created in habitats where elephants had been definitively located. Similarly, an equal number of random pseudo‐absences to the number of presences within the validation set of blocks were created. We used these balanced datasets (i.e., the same number of presence and pseudo‐absences) for model fitting and model validation at each iteration because the performance of random forest has been shown to decrease when using imbalanced datasets (Barbet‐Massin et al., 2012; Evans et al., 2017; Sillero et al., 2021). Each random forest was run with 500 trees, which surpasses the threshold beyond which there is no improvement in AUC values for similar datasets (Oshiro et al., 2012). We calculated the relative importance of each predictor variable as the averaged proportional contribution of each band, indicated by the GINI index that is calculated by each random forest classifier at each model iteration. We made separate model predictions for each of the 10 model iterations and then averaged habitat suitability index of each pixel to obtain a final habitat suitability index map.

We used ArcGIS Pro 2.8.0 (Esri Ltd.) to summarize the percent of predicted suitable habitat inside and outside Sumatra's protected areas. We considered national parks, wildlife reserves, natural reserves, grand forest parks, natural tourism parks, and grand gardens receiving conservation status by the Indonesian government as protected areas. We used the Region Group tool in ArcGIS Pro to map patches of suitable habitat based on the potential distribution map. We defined a patch as all orthogonally contiguous raster cells of suitable habitat with a total area >275 km^2^—the minimum area‐corrected autocorrelated kernel density estimated (AKDE_C_) home range size for elephants in Bukit Tigapuluh, Sumatra (Moßbrucker et al., 2016).

To assess the functional connectivity between known elephant population ranges, we used Circuitscape 4.0 (Anantharaman et al., 2019). Circuitscape uses circuit theory to model connectivity and considers the landscape as a resistance surface on which animals move as random walkers without complete knowledge of the landscape (McRae et al., 2013). The resulting resistance and conductance values are proportional, showcasing the relative probability of movement through different pathways on the landscape (Shah & McRae, 2008). We derived our resistance surface from our habitat suitability prediction, inverting the suitability values and scaling between 1 and 100 (i.e., minimum to maximum resistance). We used the program's pairwise mode, which considers each known population as an electrical node and runs a theoretical current between each pair of nodes. A cumulative connectivity map was created by adding the currents generated between every node pair. We identified corridors in the top 15% of cumulative current as areas of high connectivity (Theron et al., 2022).

### 2.3 Model Evaluation & Validation

To assess habitat suitability model accuracy, we calculated the area under the precision‐recall curve (AUC‐PR), sensitivity (the true positivity rate), and specificity (the true negativity rate) for each model iteration (Fielding & Bell, 1997; Sofaer et al., 2019). To calculate sensitivity and specificity, we used the averaged threshold value that maximized the sum of the sensitivity and specificity among the 10 model iterations. This is the recommended threshold selection method for presence‐only data as it produces a similar threshold when using presence‐only data as when using presence‐absence data (Liu et al., 2013, 2016). We also applied this threshold to create a binary potential distribution map across the island. Additionally, we validated the final habitat suitability prediction using an independent geolocation dataset for Sumatran elephants (Figure 2b). The geolocation dataset was collected from global positioning system (GPS) collars fitted on elephants by a variety of organizations in conjunction with the Indonesian Ministry of Environment and Forestry (see Tables S2 and S3). Much of these data were lacking critical supporting information (i.e., date, time, dilution of precision). To reduce potential biases from inaccurate or duplicated geolocations, we relied on data summaries for model validation. We produced a histogram to examine the habitat suitability predicted by our model at the GPS point locations, understanding that the model would be a good representation of elephant suitable habitat if collared elephants consistently used the predicted suitable habitat (i.e., pixels with HSI above the threshold value). We also calculated the sensitivity for this GPS point dataset using the averaged threshold value.

## 3 RESULTS

Our models achieved high predictive capability, with 7 out of 10 models producing AUC‐PR values >0.7 (range: 0.61–0.80, mean = 0.73). Average sensitivity was 0.76 (range: 0.71–0.84), while average specificity was 0.68 (range: 0.46–0.81). When estimating the likelihood of suitable habitat at the independent GPS collar geolocations, we found that 83.4% of geolocations had a likelihood ≥0.425—the threshold at which the sum of the sensitivity and specificity were maximized (Figure S1). GPS collar geolocations overlapped 8 of the 22 known elephant ranges on Sumatra, with 76.9% of geolocations in Bukit Tigapuluh (*n* = 115,637). Human modification contributed the most to model fitting (26%), followed by surface ruggedness (23%) and NDVI (17%; Table 3).

**TABLE 3:** Variable importance in model creation.

Layer	Random Forest importance calculation	Percent contributed
Human modification	163.118	25.62%
Surface ruggedness	145.477	22.85%
NDVI	110.210	17.31%
Sentinel Radar	100.787	15.83%
Palsar Radar	90.274	14.18%
Palm oil	26.888	4.22%

*Note*: The human modification, surface ruggedness, and NDVI layers contributed the most to model creation.

**FIGURE 3:**
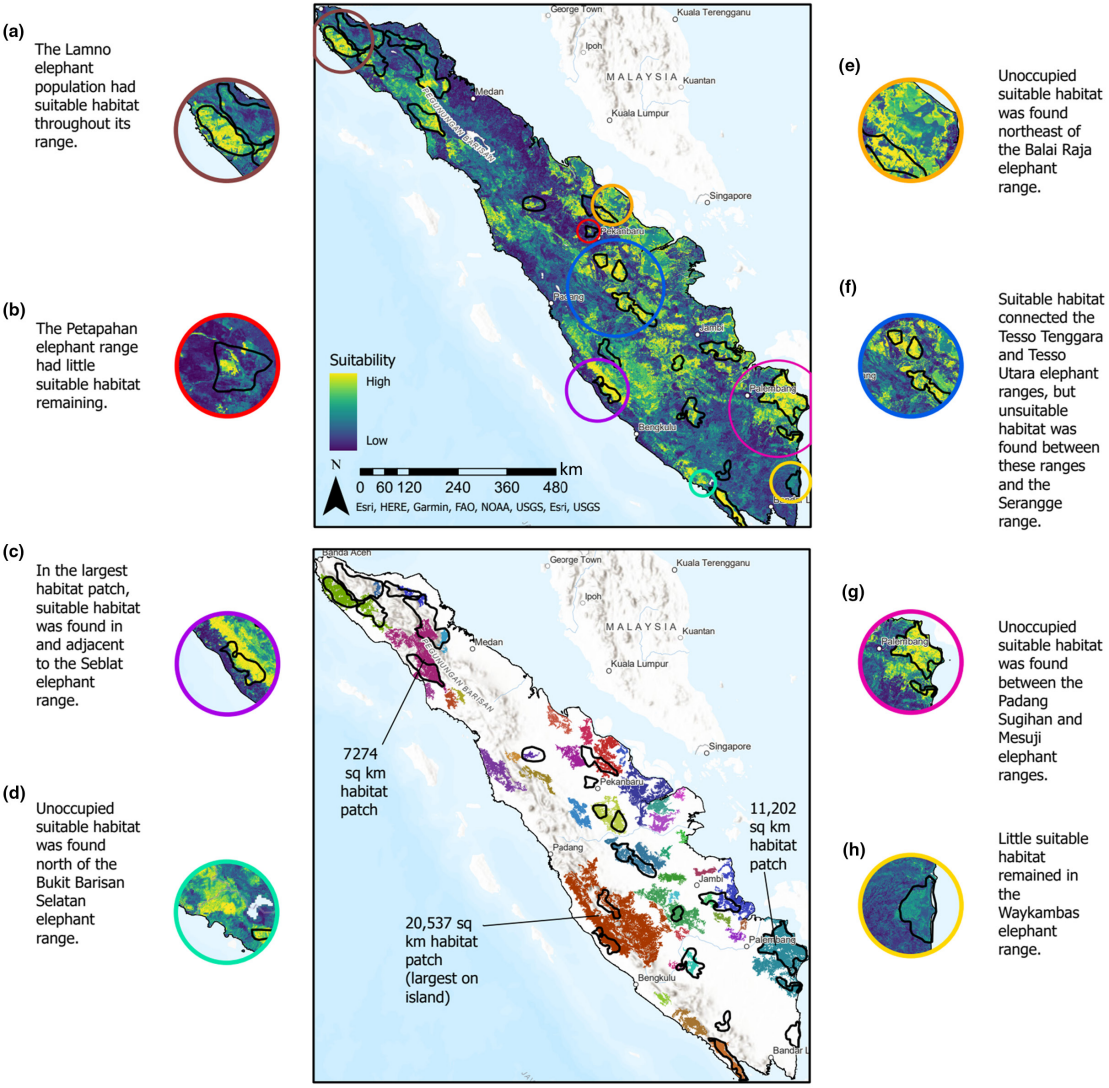
Predicted elephant habitat suitability based on the mean of the 10 model iterations (top map) and patches of suitable habitat (bottom map). The habitat patch map was created using the average threshold value that maximized the sum of sensitivity and specificity among the 10 iterations. The model suggests suitable elephant habitat is distributed in patches across the island.

Thirty‐two percent or 135,646 km^2^ of Sumatra's land area was predicted to be suitable habitat (Figure 3). Seventy‐nine percent of this habitat or 107,319 km^2^ was located outside of protected area boundaries. Sixty percent of land within protected area boundaries or 28,327 km^2^ was predicted to be suitable. Forty‐three patches of suitable habitat were found across Sumatra. The largest patch of contiguous habitat (20,537 km^2^) extended into the Bengkulu, West Sumatra, Jambi, and South Sumatra provinces and included the Sipurak and Seblat elephant ranges (Figure 3b). Other large suitable habitat patches were found in the Ogan Komering Ilir and Banyuasin regencies in the South Sumatra province (11,202 km^2^) and in the South Aceh, Southeast Aceh, Southwest Aceh, and Gayo Lues regencies in the Aceh province (7274 km^2^). Most of Sumatra's 22 elephant ranges, with the exception of Petapahan (Figure 3b), Waykambas (Figure 3h), HPT, Saka, and Seulawah, contained suitable habitat. Lamno was notable as the elephant range with most of the suitable habitat in northern Sumatra (Figure 3a). Unoccupied suitable habitat was also predicted adjacent to elephant ranges, with areas of note adjacent to Seblat (Figure 3c), north of Bukit Barisan Selatan (Figure 3d), northeast of Balai Raja, between Tesso Tenggara and Tesso Utara (Figure 3f), and between Padang Sugihan and Mesuji (Figure 3g).

**FIGURE 4:**
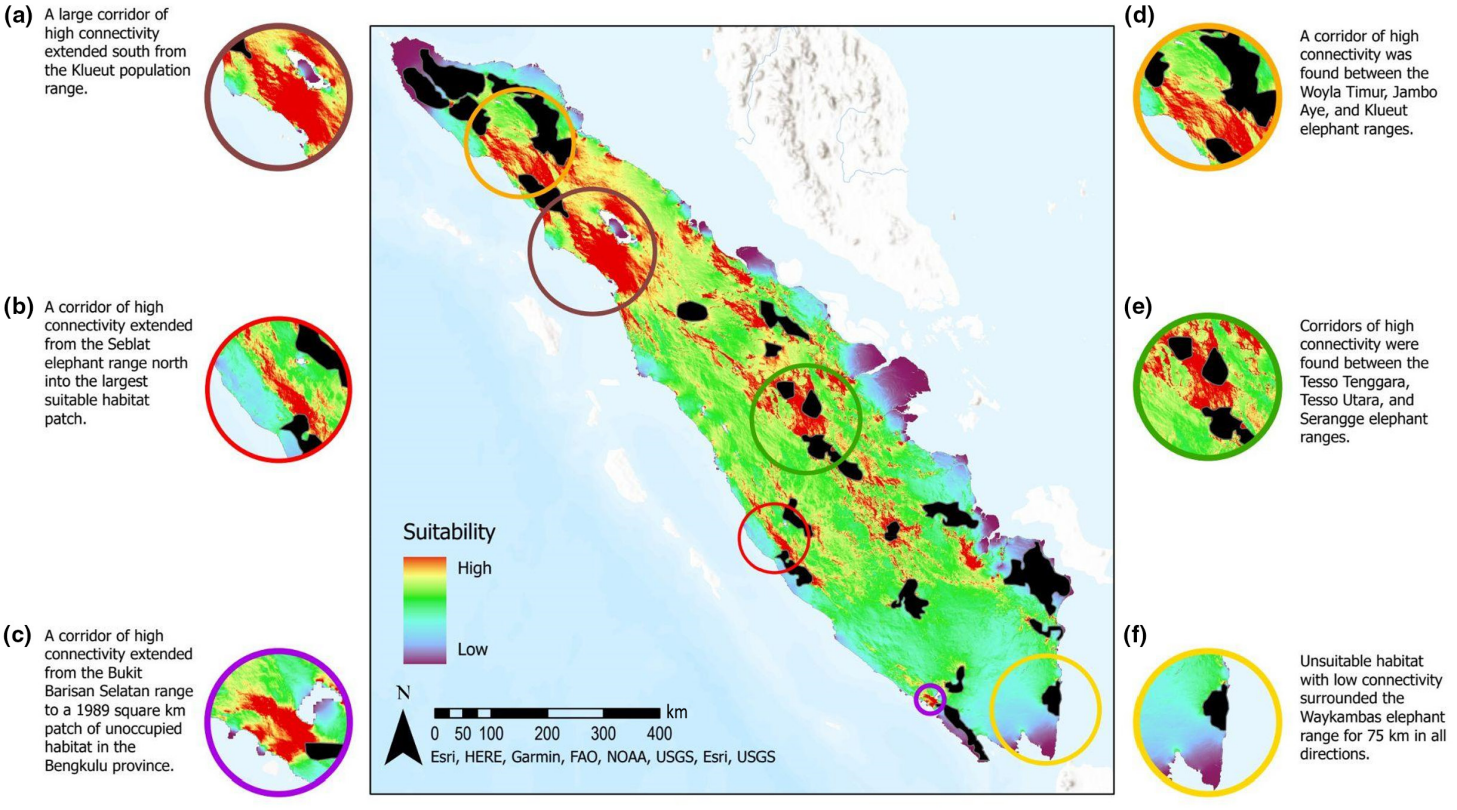
Landscape connectivity for elephants across the island of Sumatra. Areas adjacent to elephant ranges with notable connectivity are highlighted in the inset maps (a‐f). Areas with high connectivity were concentrated in the Riau and North Sumatra provinces.

Areas with high connectivity were concentrated in the Riau and North Sumatra provinces (Figure 4). Corridors of high connectivity extended south from the Klueut elephant range (Figure 4a), between the Woyla Timur, Jambo Aye, and Klueut elephant ranges (Figure 4d), and between the Tesso Tenggara, Tesso Utara, and Serangge elephant ranges (Figure 4e). A corridor of high connectivity extended from the Seblat elephant range north into the largest patch of contiguous habitat (Figure 4b), and a corridor of high connectivity also extended from the Bakut Barisan Selatan range to a 1989 km^2^ patch of adjacent, unoccupied habitat (Figure 4c). The Way Kambas range was surrounded by regions with high movement cost (Figure 4f).

## 4 DISCUSSION

Our model predicted suitable elephant habitat across all provinces in Sumatra. Thirty‐two percent of the island was suitable habitat even though only 12% had occupied elephant ranges, suggesting there is unoccupied suitable elephant habitat. Forty‐three suitable habitat patches larger than the minimum Sumatran elephant home range size were spread across the island. This remaining suitable habitat available to elephants on Sumatra likely relates to the species' adaptability to various habitats, including grasslands, open forest, and agricultural landscapes (de la Torre et al., 2021; Moßbrucker et al., 2016). Elephants are also highly intelligent and exhibit flexibility in regards to landscape and resource use (Koirala et al., 2015; Plotnik & Jacobson, 2022). Our study suggests that more suitable habitat remains on Sumatra than is used by the known populations.

Our analysis found that the landscape matrix provides roughly four times as much suitable habitat than protected areas. This is especially important since most of Sumatra's protected areas are smaller than an elephant's home range (Moßbrucker et al., 2016). Many areas with the most suitable habitat were outside of protected areas but within known elephant ranges (i.e., Lamno, Tesso Tanggara, Tesso Utara, Bukit Tigapuluh, Seblat, Padang Sugihan). Conserving this and other unoccupied habitat will likely require establishing conservation management practices on lands outside of the traditional category of protected areas. The Indonesian government Kawansan Ekosistem Esensial (KEE or Essential Ecosystem Area) initiative may provide a mechanism to conserve these additional elephant habitats. This initiative, which designates both public and private lands as KEE, has already protected four elephant movement corridors in Aceh, Bangkulu, and Jambi provinces (Tjahjana, 2020). More habitat could be protected as Indonesia plans to increase ecosystem conservation across an additional 11% of its land area, aligning the country with the Convention on Biological Diversity and the push for other effective conservation measures (OECMs; Alves‐Pinto et al., 2021; Donald et al., 2019; Sahide et al., 2020). Still, Sumatra's elephant population is frighteningly small and isolated. Habitat loss caused by land‐use change likely explains why roughly two‐thirds of the island's habitat was predicted to be unsuitable (de Silva et al., 2023).

Suitable habitat remained in and around most of the occupied elephant ranges, with notable patches by the Klueut, Balai Raja, Sipurak, Seblat, Bukit Barisan Selatan, and Padang Sugihan ranges. This immediate proximity suggests the potential for individuals from these populations to colonize the adjacent, unoccupied habitat (Metzger & Décamps, 1997; Moßbrucker et al., 2016). The strong functional connectivity extending from the Klueut elephant range into unoccupied suitable habitat south of the range implies the best available corridor to facilitate elephant movement between the Aceh and central Sumatran populations. The corridors of high connectivity between the Tesso Tenggara, Tesso Utara, and Bukit Tigapuluh elephant ranges similarly suggest potential movement of individuals among these populations. The lack of genetic connectivity in these circumstances, however, may indicate dispersal offers little benefit. This could be because the habitat in the elephant ranges is adequate to support current population numbers or that elephant movement is stymied by barriers that are not accounted for in our model (Howell et al., 2018; Mühlner et al., 2010).

Low connectivity existed between the Balai Raja population and the unoccupied suitable habitat to the northeast, suggesting that individual elephants may have difficulty colonizing this habitat despite its immediate proximity. Way Kambas National Park in southern Sumatra was strikingly predicted to be unsuitable habitat for elephants, despite hosting a large elephant population and having observations from these elephants input into our model. Given the amount of resources spent on elephant conservation in this park, this finding may suggest that areas found to be unsuitable by our analysis are able to support elephants if efforts are applied to minimize human encroachment. Way Kambas National Park was also surrounded by unsuitable habitat with low connectivity in all directions for at least 75 km. The isolation of the park questions if the population could reach a habitat‐limited carrying capacity (Odum, 1953) and/or genetic isolation (Goossens et al., 2016). The little habitat remaining in the Mahato range suggests this population may also be in danger of further decline (Gopala et al., 2011).

## 5 MODEL LIMITATIONS

The quality of our species distribution model depends on the quality of the input data (Araújo et al., 2019; Leroy et al., 2018; Sillero et al., 2021). The accuracy metrics of both the observational presence geolocation and independent GPS collar geolocation datasets suggest our model is robust. However, we had no observational data from 10 out of the 22 known elephant populations, with 67% of our observations originating from four elephant ranges. The GPS geolocation data had a similar geographic bias, corresponding to only eight elephant ranges. Both the observational and GPS collar datasets had the most geolocations in the Bukit Tigapuluh range, suggesting that our independent GPS data may be partially validating the skewness in the locations of the observational data within the habitat suitability prediction. Many of our observations were also located on roads, with 10 random samples of 100 random observation points resulting in an average of 18 observations on roads. This potentially represents a sampling error associated with observer bias toward roads and the failure of observers to document the observed elephant's location rather than their own. Elephants, however, are known to select for open habitat and secondary forest found near roads (Roever et al., 2013; Wadey et al., 2018). We encourage all observers to take care to document the exact geolocations of elephants seen in the field to improve the relationship with environmental covariates and results.

The human modification layer, which contributed the most to model fitting, likely does not quantify all human activities that impact elephant spatial distribution. More precise mapping of human land use would improve the accuracy of our model. Biological interactions with additional species, such as fruiting trees (Benitez & Queenborough, 2021), were also not captured by our model but likely influence Sumatran elephant distribution (Mpakairi et al., 2017; Wisz et al., 2013). The temporal dynamics of these effects were also not accounted for in our spatial modeling framework.

## 6 CONSERVATION IMPLICATIONS & NEXT STEPS

With so few Sumatran elephants remaining in the wild, there is a demand for accurate maps to inform conservation activities (Suhartono et al., 2007). We integrated the best available data on Sumatran elephant occurrence to identify the areas that provide suitable habitat for the species. The amount of suitable habitat remaining, which includes grassland, open forest, and agricultural land types, suggests that opportunities for targeted conservation still exist. Deforestation and increased intensity of human land use negatively impact elephant habitat use (Jathanna et al., 2015; Kumar et al., 2010; Rood et al., 2010). Efforts to reduce the intensity of human land use and prevent land conversion of elephant habitat should focus on the areas identified as highly suitable, especially in or near existing elephant ranges. Efforts should likewise prioritize the areas found to be corridors of high landscape connectivity, which will increase the probability of genetic connectivity among the remaining elephant population fragments (Bandara, 2020; Christie & Knowles, 2015). On Sumatra, areas designated as protected have lower deforestation rates than unprotected areas sanctioned for conservation, but similar deforestation rates to unprotected production areas where commercial logging is sanctioned but land conversion is not (Gaveau et al., 2012). The focus for the areas identified as highly suitable and/or of high landscape connectivity should therefore be on preventing land conversion, with protected area designation prioritized if the necessary financial resources and personnel are available. We encourage local and central government officials to integrate this spatial information into land planning efforts.

Improving the habitat suitability model will require future research to focus on improving the quality of data collected in the field. We recommend the creation of a central government repository for Sumatran elephant data, as well as increased data sharing among the relevant government agencies and non‐profit organizations. Rather than simply collecting data from known elephant ranges, we recommend using the results from this study to identify areas in which to focus new sampling efforts. Especially useful may be data collected across environmental gradients, which would allow future analyses to pinpoint critical thresholds in these variables beyond which elephant occurrence may be limited.

Data collected to model the occupancy of elephants via a coordinated camera trap grid would assess the relative probability of detection (MacKenzie et al., 2017; Widodo et al., 2022) and improve knowledge of elephant distribution. A central question that remains unanswered is why elephants do not occupy the suitable habitat adjacent to known ranges. Understanding further detail about how human presence on the landscape affects elephant distribution may allow elephants to disperse into this unoccupied habitat. The rapid and ongoing conversion of Sumatra's forest to agriculture and silviculture also remains a major concern.

The rapid decrease in elephant abundance demonstrates that the subspecies is at a crisis point and that conservation action is crucial to maintaining the wild elephant presence on the island. Our results suggest that conservation management on key habitat patches and corridors may help the endangered Sumatran elephant persist in situ. We encourage all stakeholders to scale‐up efforts to conserve the subspecies so population numbers may once again grow.

## AUTHOR CONTRIBUTIONS

**Muhammad Ali Imron:** Conceptualization (lead); data curation (lead); formal analysis (supporting); project administration (equal); resources (lead); supervision (lead); writing – original draft (lead); writing – review and editing (equal). **Danielle M. Glass:** Data curation (equal); formal analysis (lead); methodology (equal); validation (equal); visualization (lead); writing – original draft (lead); writing – review and editing (equal). **Muhammad Tafrichan:** Conceptualization (lead); data curation (lead); formal analysis (supporting); project administration (lead); writing – review and editing (supporting). **Ramiro D. Crego:** Formal analysis (equal); methodology (lead); software (equal); validation (equal); visualization (supporting); writing – review and editing (equal). **Jared A. Stabach:** Conceptualization (supporting); formal analysis (equal); funding acquisition (equal); methodology (equal); project administration (equal); supervision (equal); writing – original draft (supporting); writing – review and editing (equal). **Peter Leimgruber:** Conceptualization (supporting); formal analysis (equal); funding acquisition (equal); methodology (equal); project administration (equal); supervision (lead); writing – original draft (supporting); writing – review and editing (supporting).

## FUNDING INFORMATION

Funding for this publication was provided by the Smithsonian Institution's Movement of Life Initiative and Universitas Gadjah Mada.

## Supporting information


Appendix S1

## Data Availability

We have included the Google Earth Engine script used in the suitability analysis, the R code used in the connectivity analysis, and the habitat suitability shapefile in the Supporting Information. All authors and partner organizations are in agreement to not share the underlying geolocation data with the public at <50 km resolution due to the critically endangered status of the species.
