# Supplementary material for: Beyond protected areas: The importance of mixed‐use landscapes for the conservation of Sumatran elephants (Elephas maximus sumatranus)
Source: Ecol Evol. 2023 Sep 28;13(10):e10560. doi: 10.1002/ece3.10560 (PMC10539044; doi:10.1002/ece3.10560)
Supplement: Supplementary file 1 — Appendix S1 [file ECE3-13-e10560-s001.docx]

**SUPPORTING INFORMATION**

5 August 2023

Muhammad Ali Imron^*^, Danielle M. Glass^*^, Muhammad Tafrichan, Ramiro D. Crego, Jared A. Stabach, Peter Leimgruber. Beyond Protected Areas: The importance of mixed-use landscapes for the conservation of Sumatran elephants (*Elephas maximus sumatranus*).

* Both authors have contributed equally

The Google Earth Engine code used to generate the species distribution model can be found below. Note that the elephant geolocation data has been redacted due to ethical and third party restrictions. See the Data Accessibility Statement for more information.

<https://code.earthengine.google.com/b3ff5fe110b4972045f1ccd599770207>

For looking at the correlation between the environmental variables:

<https://code.earthengine.google.com/9f2e734026ece0a0cec399acc713e3e2>

For the Dryad repository:

[https://datadryad.org/stash/share/YGl2KM5cTXLCfN2GyDCseSPjv6IjDmzF43AXDuzZ8gQ](https://nam02.safelinks.protection.outlook.com/?url=https%3A%2F%2Fdatadryad.org%2Fstash%2Fshare%2FYGl2KM5cTXLCfN2GyDCseSPjv6IjDmzF43AXDuzZ8gQ&data=05%7C01%7Cglassd%40si.edu%7Cb0cf01cfc86a452ea0f808db4ac38f8e%7C989b5e2a14e44efe93b78cdd5fc5d11c%7C0%7C0%7C638185976031617789%7CUnknown%7CTWFpbGZsb3d8eyJWIjoiMC4wLjAwMDAiLCJQIjoiV2luMzIiLCJBTiI6Ik1haWwiLCJXVCI6Mn0%3D%7C3000%7C%7C%7C&sdata=V%2FTaWLRg2z7gHveJNSAHpoCxmhH4uYM75jsSyOlUseU%3D&reserved=0)

Table S1. Elephant observations by data source. Observations are divided between direct observations of elephants and observations of elephant signs. Ultimate data source refers to the organization that collected the data, while proximate data source refers to the organization or institution from which the data was obtained.

| **Ultimate Data Source** | **Proximate Data Source** | **Number of Direct Observations** | **Number of Elephant Sign Observations** |
| --- | --- | --- | --- |
| South Sumatra Natural Resources Conservation Agency, Indonesian Ministry of Environment and Forestry | South Sumatra Natural Resources Conservation Agency, Indonesian Ministry of Environment and Forestry | 2 | 462 |
| PT. Restorasi Ekosistem Indonesia (a consortium of Burung Indonesia, Birdlife International, and The Royal Society for the Protection of Birds working in the Hutan Harapan ecosystem restoration concession) | South Sumatra Natural Resources Conservation Agency, Indonesian Ministry of Environment and Forestry | 1 | 0 |
| Directorate of Biodiversity Conservation, General Directorate of Natural Resource Conservation, the Indonesian Ministry of Environmental and Forestry | Directorate of Biodiversity Conservation, General Directorate of Natural Resource Conservation, Indonesian Ministry of Environment and Forestry | 0 | 69 |
| Bukit Barisan Selatan National Park | Directorate of Biodiversity Conservation, General Directorate of Natural Resource Conservation, Indonesian Ministry of Environment and Forestry | 223 | 266 |
| Riau Natural Resources Conservation Agency, Indonesian Ministry of Environment and Forestry | Directorate of Biodiversity Conservation, General Directorate of Natural Resource Conservation, Indonesian Ministry of Environment and Forestry | 0 | 731 |
| Taman Nasional Gunung Leuser (Leuser Mountain National Park) | Directorate of Biodiversity Conservation, General Directorate of Natural Resource Conservation, Indonesian Ministry of Environment and Forestry | 0 | 225 |
| Jambi Natural Resources Conservation Agency, Indonesian Ministry of Environment and Forestry | Jambi Natural Resources Conservation Agency, Indonesian Ministry of Environment and Forestry | 0 | 973 |

Table S2. Elephant GPS geolocation data by data source. Ultimate data source refers to the organization that collected the data, while proximate data source refers to the organization or institution from which the data was obtained. For the Frankfurt Zoological Society dataset, the number of geolocations by individual represents the number after the removal of geolocations earlier or equal to the day of collar placement.

| **Ultimate Data Source** | **Proximate Data Source** | **Number of collared individuals** | **Time periods that collars were active** | **Number of geolocations by individual** |
| --- | --- | --- | --- | --- |
| Bukit Barisan Selatan National Park | Directorate of Biodiversity Conservation, General Directorate of Natural Resource Conservation, Indonesian Ministry of Environmental and Forestry | 3 | For 2 individuals exact dates unknown but in the year of 2020, for 1 individual exact dates unknown but in the year 2019 | - 97 - 2417 - 3291 |
| Aceh Natural Resources Conservation Agency, Indonesian Ministry of Environment and Forestry | Directorate of Biodiversity Conservation, General Directorate of Natural Resource Conservation, Indonesian Ministry of Environmental and Forestry | 2 | For 1 individual exact dates unknown but in the time period June - August 2019, for 1 individual exact dates unknown but in the years 2017-2018 | - 3226 - 209 |
| Bukit Barisan Selatan National Park | Directorate of Biodiversity Conservation, General Directorate of Natural Resource Conservation, Indonesian Ministry of Environmental and Forestry | 1 | Unknown time period in 2018, 1/2020-1/2021 | - 1218 |
| Riau Natural Resources Conservation Agency, Indonesian Ministry of Environment and Forestry | Directorate of Biodiversity Conservation, General Directorate of Natural Resource Conservation, Indonesian Ministry of Environmental and Forestry | 3 | Exact dates unknown for all individuals. One collar documented geolocations in 2013, one in 2015, and one in 2020. | - - 1880 - - 586 - - 2096 |
| Jambi Natural Resources Conservation Agency, Indonesian Ministry of Environment and Forestry | Jambi Natural Resources Conservation Agency, Indonesian Ministry of Environment and Forestry | 16 | Collars active 2012-2020. See Table S3 for exact dates. | - 14942  - 1908  - 20190  - 8688  - 569  -19399  - 18241  - 17838  - 16343  - 3447  - 3934  - 3648  - 2286  - 2711  -1388  - 500 |

Table S3. Fix rate and total number of geolocations for the 16 collared elephants in the Jambi Natural Resources Conservation Agency’s dataset. The total number of geolocations is the number of observations after removing geolocations on or before the day of collar placement on the elephant. Jenny 1 and Jenny 2 are different elephants.

| **Elephant Identification** | **Total Number of GPS Geolocations** | **Collar Fix Rate by Time Period** |
| --- | --- | --- |
| Anna | 14942 | - 7/26/12 - 9/16/13 4 hr fix rate - 9/16/13-9/18/17 2 hr fix rate - 9/18/17-11/30/17 fixes at 3 am, 11 am, 19 pm |
| Bella | 1908 | - 7/30/12-6/16/13 4 hr fix rate |
| Cinta | 20190 | - 7/31/12-1/26/14 4 hr fix rate - 1/26/14-9/17/17 2 hr fix rate - 9/17/17-3/19/18 8 hr fix rate - 3/22/18-3/28/18 1 hr fix rate - 3/28/18-1/11/19 8 hr fix rate - 1/11/19-6/25/19 every hour at :03 and :34 - 6/25/19-7/14/19 every hour at :03 and :34, also every 3 hr starting at 00:00 am - 7/14/19-4/2/20 3 hr fix rate |
| Dadang | 8688 | - 8/1/12-9/16/13 4 hr fix rate - 9/16/13-1/28/16 2 hr fix rate |
| Elena | 569 | - 8/4/12-12/28/13 4 hr fix rate |
| Freda | 19399 | - 7/31/13-12/13/13 4 hr fix rate - 12/13/13-1/26/14 2 hr fix rate - 1/26/14-2/7/14 1 hr fix rate - 2/7/14-3/13/15 2 hr fix rate - 3/14/15 - 8/23/16 2 hr few second fix rate - 8/26/16-9/24/17 2 hr fix rate - 9/25/17-7/8/19 4 hr fix rate - 7/8/19-4/2/20 3 hr fix rate |
| Ginting | 18241 | - 1/25/14-9/18/17 2 hr fix rate - 9/18/17-3/21/18 8 hr fix rate - 3/23/18-4/2/20 3 hr 3 min fix rate |
| Haris | 17838 | - 11/16/14-9/6/16 2 hr fix rate - 9/6/16-9/12/16 1 hr fix rate - 9/13/16-9/9/17 2 hr fix rate - 9/17/17-4/11/18 1 hr 1 min fix rate - 4/11/18-9/25/19 3 hr 1 min fix rate |
| Indah | 16343 | - 1/10/15-9/24/17 2 hr fix rate - 9/24/17-7/12/19 4 hr fix rate - 7/12/19-7/20/19 1 hr fix rate - 7/20/19-4/2/20 3 hr fix rate |
| Jenny 1 | 3447 | - 1/19/16-6/5/17 2 hr fix rate |
| Jenny 2 | 3934 | - 9/17/17-3/26/19 4 hr fix rate - 3/26/19-7/28/19 3 hr fix rate |
| Karina | 3648 | - 10/11/16-9/21/17 2 hr fix rate - 9/21/17-1/24/19 1 hr fix rate |
| Lucky | 2286 | - 10/6/18-1/20/20 3 hr 3 min fix rate |
| Mutiara | 2711 | - 2/17/19-7/8/19 4 hr fix rate - 7/8/19-4/2/20 3 hr fix rate |
| Nikolas | 1388 | - 9/30/19-4/2/20 3 hr fix rate |
| Ozzy | 500 | - 3/11/20-4/2/20 1 hr 2 min fix rate |

**
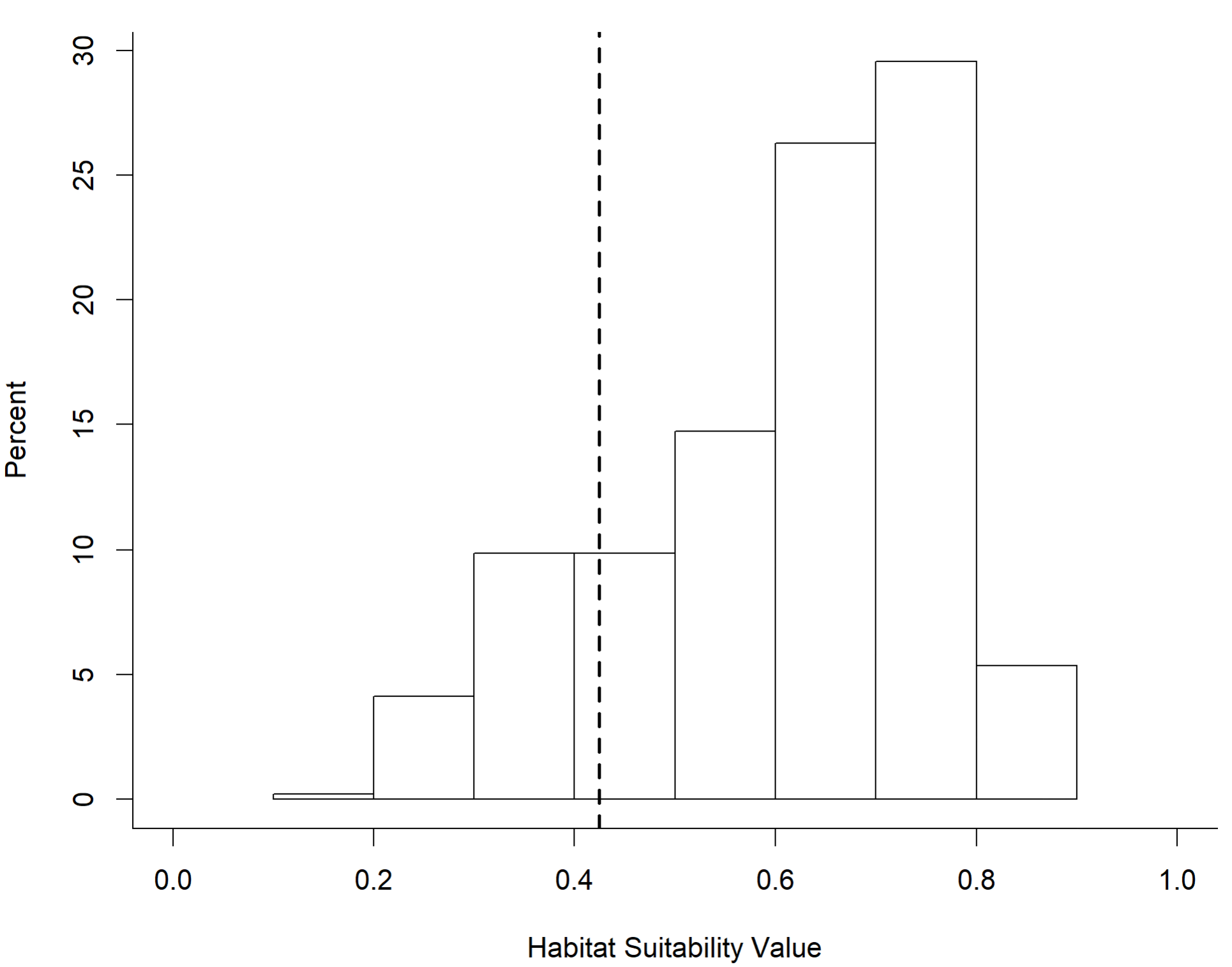
**

Figure S1. Habitat suitability at geolocations from the independent GPS collar geolocation dataset. The vertical dotted line represents the averaged threshold value that maximized the sum of the sensitivity and specificity among the 10 model iterations. Our random forest model suggests the vast majority of geolocations were located in suitable elephant habitat.
